# The Advanced Integrated Respiratory (AIR) Model: Comparative Analysis of Salbutamol Sulphate Deposition from pMDI, DPI, and Nebuliser Versus the NGI

**DOI:** 10.1007/s11095-026-04036-0

**Published:** 2026-02-20

**Authors:** Patrick He, Hazel Lam, Damien Chong, Shaokoon Cheng, Patrick Spicer, Paul Michael Young, Lois Ledo, Daniela Traini, Hui Xin Ong

**Affiliations:** 1 Respiratory Technology, Woolcock Institute of Medical Research, Macquarie University, Sydney, NSW Australia; 2 Macquarie Medical School, Faculty of Medicine, Health and Human Sciences, Macquarie University, Sydney, NSW Australia; 3 Defence Science and Technology Group, Fishermans Bend, VIC Australia; 4 School of Chemical Engineering, University of New South Wales, Kensington, NSW Australia; 5 Macquarie Business School, Macquarie University, Sydney, NSW Australia; 6 School of Engineering, Macquarie University, Sydney, Australia

**Keywords:** aerosol delivery platform, DPI, *in vitro* lung model, nebuliser, PMDI

## Abstract

**Purpose:**

*In vitro* respiratory models such as the Next Generation Impactor (NGI), remain the gold standard for aerodynamic particle size distribution (APSD) testing, however, they lack the anatomical complexity, limiting their ability to replicate *in vivo* deposition. To address this limitation, the Advanced Integrated Respiratory (AIR) model, a physiologically relevant benchtop system incorporating anatomically accurate silicone casts of the upper and lower airways, was used to assess deposition of salbutamol sulphate delivered via three clinically relevant platforms.

**Methods:**

Salbutamol sulphate was delivered using a pressurised metered-dose inhaler (pMDI), a dry powder inhaler (DPI), and a jet nebuliser. Deposition profiles obtained in the AIR model were benchmarked against the NGI.

**Results:**

Both systems showed consistent patterns for DPI and nebuliser aerosols, with highest deposition in the oropharyngeal and intrathoracic regions, respectively. In contrast, pMDI testing revealed important differences: the AIR model predicted markedly higher oropharyngeal retention and reduced intrathoracic delivery, aligning more closely with published *in vivo* scintigraphy studies than the NGI.

**Conclusion:**

These findings demonstrate that anatomically realistic models provide critical insights into deposition behaviour, particularly for propellant driven inhalers and underscore the value of integrating physiologically relevant platforms alongside conventional impactors in aerosol characterisation.

## Introduction

Deposition of inhaled drugs is influenced by the aerodynamic properties of the generated aerosol, among which particle size is the dominant determinant of regional deposition within the respiratory tract [1]. Aerosol behaviour in the airways is controlled by three main mechanisms of inertial impaction, gravitational sedimentation, and Brownian diffusion [2]. Particles with > 5 µm typically deposit in the oropharynx via inertial impaction, where they are likely swallowed, resulting in low pulmonary delivery and potential systemic side effects through gastrointestinal absorption [3]. Whereas particles between 1–5 µm are more likely to penetrate the small airways and achieve therapeutic efficacy via gravitational sedimentation [4]. Submicron particles offer pharmacological benefits for pulmonary delivery as they reach the deep lung through Brownian diffusion [5], however, they are also prone to exhalation before deposition [4]. Consequently, accurate characterisation of aerosol particle size and deposition pattern is important to the development, safety, and clinical performance of inhaled therapeutics.

*In vitro* aerosol characterisation is hence a crucial assessment for inhalation product development. Cascade impactors, most notably the Next Generation Impactor (NGI), remain the gold standard for *in vitro* evaluation of aerodynamic particle size distribution (APSD) [6]. NGI assess aerosols fractionated across a series of size-selective stages dependent on flow rate, allowing quantification of emitted dose and fine particle fraction (FPF) [6, 7], APSD data derived from the NGI are widely used for predicting pulmonary deposition and supporting quality control of formulations and devices [6, 8]. However, the non-anatomically accurate NGI lacks the geometric complexity to accurately assess the regional deposition pattern.

Among delivery platforms, the pressurised metered-dose inhaler (pMDI) is the most widely used for salbutamol sulphate administration owing to its effectiveness, portability, low cost, and ease of use [5, 9]. Hydrofluoroalkane (HFA)-propelled pMDIs can generate aerosol particles with mass median aerodynamic diameters of ~ 1.3 µm, with up to 35% of emitted particles below 5 µm [10]. However, correct coordination of inhalation to actuation technique is important for optimal drug delivery [10]. Even with correct inhalation technique, HFA-based pMDIs tested at a flow rate of 30 L/min retain approximately 45–60% of the emitted dose in the throat, depending on the respiratory model used [11–14].

By contrast, dry powder inhalers (DPIs) are breath-actuated systems that eliminate the need for propellants [15]. Formulations are typically produced either as agglomerated micronised drug (< 5 µm) or as drug particles adhered to lactose carriers e.g. salbutamol sulphate DPI formulations [15]. The energy generated from the patient’s inhalation is used for drug powder dispersion, separation from the carrier if required and delivery into the deep lungs of the patient [15, 16]. This mitigates the inhalation and actuation coordination issue faced by patients when using pMDIs [16]. Nevertheless, lung deposition from DPIs is influenced by device resistance, patient inspiratory flow rate, and aerosol physicochemical properties [17]. With *in silico* models having shown that DPI deliver a larger dose to the mouth-throat (70%) in comparison to the MDI where 50% is delivered to the alveolar airways [18].

Unlike pMDIs and DPIs, the use of a nebuliser involves the dispersion of an aqueous formulation containing salbutamol sulphate into aerosol droplets [19]. When using a nebuliser, the patient is only required to take quiet breaths negating the need to generate significant inspiratory efforts [20]. Thus, it is mainly recommended for use by children and older patients with cognitive and physical impairments [20]. However, nebulisers require considerably longer times to administer the drug and higher doses than pMDIs and DPIs [21]. They can be viewed as inefficient and inaccurate systems of drug delivery as approximately 20% of the original dose is available for inhalation, and of this, 2–19% is deposited in the lungs [22]. These values are also dependent on the type of nebuliser system and breathing pattern of the user [22].

To bridge the gap between *in vitro* and *in vivo* assessment of respiratory deposition, 3D printed realistic airway models have been in development [23–25]. The Advanced Integrated Respiratory (AIR) Model is a bench-top system designed to replicate respiratory aerosol deposition under physiologically relevant conditions. It was developed to address limitations of traditional *in vitro* respiratory models, which often lack anatomical structural complexity. The AIR model incorporates a 3D cast of the respiratory tract comprising silicone-based upper and lower airways, representing the trachea and bronchi, with lower airway is encased within a Plexiglass® chamber to mimic the lung environment. Integrating anatomical complexity with controlled experimental conditions, the AIR model aims to complement established impactor models.

In this study, the AIR model was characterised by evaluating deposition profiles of salbutamol sulphate delivered via three clinically relevant inhalation platforms, including DPI, pMDI, and nebuliser. These formulations were then benchmarked against the NGI. Salbutamol sulphate, a frontline bronchodilator widely prescribed for the management of asthma and chronic obstructive pulmonary disease, was selected as a model compound [6]. This comparative approach allows for assessment of how formulation and device-specific aerosol characteristics translate into deposition behaviour under physiologically relevant conditions.

## Materials and Methods

### Materials

Salbutamol sulphate was obtained from three commercially available inhalation products: Ventolin® CFC-free pressurised metered-dose inhaler (pMDI) with dose counter (100 µg per actuation, GlaxoSmithKline, UK), Ventolin® Nebules (5 mg/2.5 mL, GlaxoSmithKline, UK), and Easyhaler® dry powder inhaler (100 µg per actuation, Orion Pharma Ltd, Finland). All solvent and chemicals used were of analytical grade (Sigma Aldrich, USA).

### Assembly of the AIR Model

**Fig. 1:**
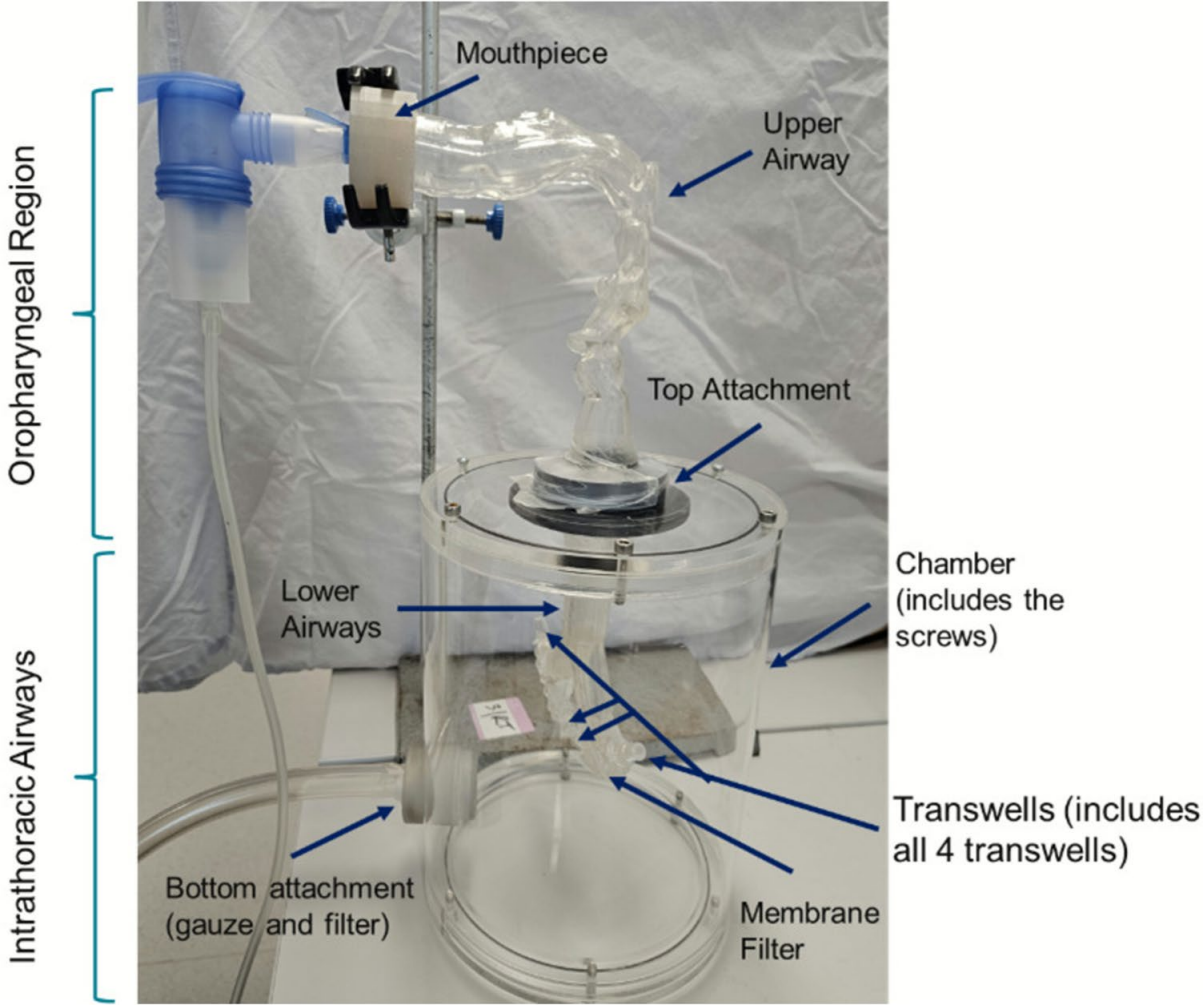
The components of the AIR model which were washed and samples collected. The oropharyngeal region includes the mouthpiece, upper airway, and top attachment. The Intrathoracic airways include the lower airways, 4 transwells, membrane filter, chamber (with screws), and the bottom attachment (gauze and filter).

An anatomically representative Advanced Integrated Respiratory (AIR) model was used to evaluate regional deposition of salbutamol sulphate aerosols as shown in Fig. 1. The anatomical design of the AIR model is based on a realistic representation of the human respiratory tract. Readers are referred to part A “The Advanced Integrated Respiratory (AIR) Model: Integration of Air–Liquid Interface Cell Cultures within a Human Airway Model for Inhalation Toxicology” for a comprehensive description of the model geometry and its anatomical correlation with human airway structures. A vacuum chamber was connected to a vacuum pump (Westech Scientific Instruments, UK), with a microfiber filter (Sigma-Aldrich, USA) positioned at the outlet to capture any aerosol escaping through the vacuum port. Four Transwell inserts (0.33 cm^2^ surface area, 0.4 µm pore size; Corning Costar, USA) were attached to the terminal regions at both ends of the lower airway compartment. The remaining lower airway openings were fitted with pore-size matched membrane filters (0.4 µm pore size; Whatman, UK), and membrane filters were also applied at the Transwell attachment notches, to equalise flow resistance across all terminal branches and minimise airflow asymmetry introduced by Transwell integration. The lid of the vacuum chamber was secured to the lower airway component and then connected to the upper airway segment. A mouthpiece was attached to the top of the upper airway and supported with a laboratory clamp stand to maintain anatomical alignment and avoid torsion in the airway path. A calibrated mass flow meter (Model 4040, TSI Precision Measurement Instruments, Germany) was connected at the mouthpiece inlet, and airflow was adjusted to 15, 30, or 60 L/min using the vacuum pump.

### Aerosol Characterisation of Salbutamol Sulphate in the AIR Model

Salbutamol sulphate deposition within the AIR model was evaluated using three delivery platforms: nebuliser, pMDI, and DPI. Flow rate parameters were selected for each device based on physiologically relevant adult inspiratory ranges and established values reported in pharmacopoeia guidelines and prior studies [26, 27]. Nebulised delivery, 2 mL of salbutamol sulphate solution (5 mg/2.5 mL; total dose = 4000 µg Ventolin® Nebule) was transferred into a PARI LC® Sprint jet nebuliser, which was connected to the AIR model and operated at an airflow of 15 L/min [28] using a calibrated flow meter (Westech Scientific Instruments, Bedfordshire, UK) for 5 min. Post-nebulisation, 0.5–1 mL of the loaded salbutamol sulphate solution was left in the nebuliser chamber as residual dose.

For pMDI testing, the Ventolin® inhaler was shaken vigorously for 5 s and primed by actuating 5 doses to waste. Subsequently, 8 actuations (total dose = 800 µg) were administered into the AIR model, with each dose fired over a 6-s interval at a flow rate of 30 L/min [29] using a calibrated flow meter. A rest period of 30 s was maintained between actuations, and the inhaler was re-shaken between each dose to allow temperature equilibration and avoid freezing of the actuator nozzle.

For DPI testing, the Easyhaler® was shaken vigorously for 5 shakes and primed with 5 actuations to waste prior to testing. Unlike conventional DPIs such as Spiromax® and Turbuhaler®, which are not shaken before use, the Easyhaler® is specifically designed to be shaken prior to actuation. Eight actuations (total dose = 800 µg) were fired into the AIR model at a flow rate of 60 L/min [30] using a calibrated flow meter, with 10-s rests and re-shaking between each actuation.

Following aerosol delivery, each component of the AIR model was carefully rinsed with a 30:70 (v/v) mixture of methanol and water. The samples were collected in appropriately sized volumetric flasks for subsequent analysis by high-performance liquid chromatography (HPLC) using a validated method.

### Aerosol Characterisation of Salbutamol Sulphate by Next Generation Impactor (NGI)

The NGI (Copley Scientific, UK) was used to study the delivery of the aerosolized salbutamol sulphate to different regions of the lungs based on the aerodynamic diameter of the aerosol particles. The airflow rate was set identically for both the NGI and AIR model to enable direct comparison of aerosol deposition from liquid formulations delivered using a PARI LC® Sprint jet nebuliser (15L/min) [28], dry powder formulations delivered via EasyHaler® (30L/min) [29], and pressurised metered-dose formulations delivered via a Ventolin pMDI (60L/min) [30]. 2 mL salbutamol sulphate nebules (2 mg/mL) was transferred to a PARI LC® Sprint jet nebuliser and the solution was aerosolized into the NGI at 15 L/min [28] for 5 min. After shaking the pMDI vigorously for 5 s and priming 5 shots to waste, 8 actuations (total dose = 800 µg) were fired into the NGI for 6 s at 30L/min [29] using a calibrated flow meter, with a 30 s rest between shots and re-shaking to allow temperature equilibration and preventing the pMDI nozzle from freezing. Salbutamol sulphate DPI was assessed, to ensure efficient particle capture and prevent inter-stage losses due to particle bounce, the stages and United States Pharmacopeia (USP) throat of NGI were coated with Brij 35: glycerol: ethanol (10:50:40 v/v/v). An NGI Pre-separator was employed to capture the large lactose carrier particles from the salbutamol sulphate DPI, simulating their deposition within the oropharynx during normal inhalation [31]. After shaking the DPI vigorously for 5 shakes and primed with 5 actuations to waste, 8 actuations (total dose = 800 µg) were fired into the AIR model for 6 s, with a 10 s rest and re-shaking between shots, with an airflow of 60 L/min [30] using a calibrated flow meter. Each NGI part was thoroughly rinsed with methanol and water at a 30:70 (v/v) ratio and transferred into appropriate volumetric flasks for analytical analysis by HPLC using a validated method.

### Quantification of Salbutamol Sulphate by High Performance Liquid Chromatography

The HPLC system equipped with SPD-20A UV–VIS detector, a LC-20AT liquid chromatography, a SIL-20A HT sampler (Shimadzu, Kyoto, Japan) and a Phenomenex 00F-4252-E0 Luna 5 µm C18(2) 100 Å, LC Column (5 μm, 150 × 4.6 mm, Phenomenex, CA, USA). The mobile phase was methanol and 0.1 M NaH_2_PO_4_ and pH adjusted to 3.35 with H_3_PO_4_ at a 30:70 (v/v) ratio. The flow rate was set at 0.8 mL/min, and an injection volume of 10 μL. Retention time ~ 3 min. Standard concentrations produced linearity of R1 = 1.

### Statistical Analysis

All results are presented as the mean ± standard deviation of three independent experiments. Statistical analysis was by one-way analysis of variance (ANOVA) with Bonferroni’s multiple comparisons using GraphPad Prism 10.4.2 (GraphPad Prism Inc., La Jolla, CA, USA).

## Results

### Comparison between AIR model and NGI

**Fig. 2:**
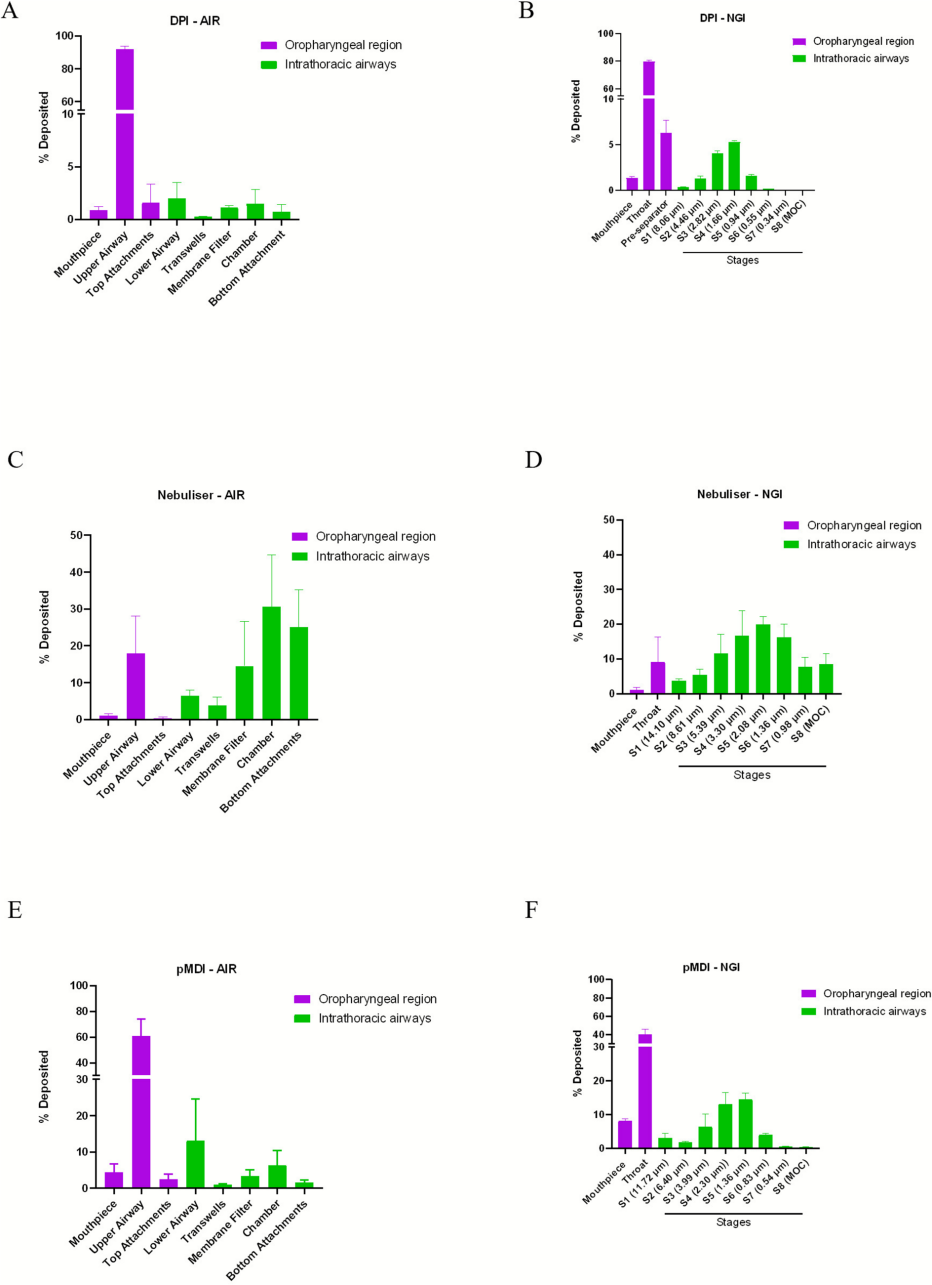
Deposition profile of salbutamol sulphate delivered via DPI, nebulisation, and pMDI. (**A**, **B**) An Easyhaler® DPI was actuated 8 times (total nominal dose: 800 µg) at a flow rate of 60 L/min, and the resulting aerosol was characterised in both the AIR model and the NGI. (**C**, **D**) A 2 mL salbutamol sulphate nebule (5 mg/2.5 mL; total nominal dose: 4000 µg) was aerosolised using a PARI LC® Sprint jet nebuliser operated at 15 L/min, with deposition quantified in the AIR model and NGI. (**E**, **F**) Ventolin® CFC-free pMDI was actuated 8 times (total nominal dose: 800 µg) at a flow rate of 15 L/min, and deposition was determined in both the AIR model and NGI. For DPI and nebuliser delivery, the actuator/device was not included in the analysis, however, pMDI actuators were washed and analysed (n = 3, mean ± SD).

The AIR model demonstrated clear separation between the oropharyngeal and intrathoracic airways, in contrast to the NGI, which segregates particles solely by aerodynamic size and velocity without anatomical constraints [32]. To evaluate model performance, we compared the deposition profile of salbutamol sulphate delivered via DPI in both systems. Both systems showed predominant throat deposition (Fig. 2A and B). In the NGI, the second highest deposition was observed in the pre-separator, which mimics the natural deposition of large particles in the oropharyngeal region during inhalation [31]. Quantitative deposition data for oropharyngeal and intrathoracic regions across all devices and models are summarised in Table I. Across the NGI stages, most particles deposited on stages 2–5 (12%), corresponding to the respirable size (1–5 µm). In contrast, the AIR model showed lower overall deposition in the intrathoracic airways, with ~ 6% detected in both the respirable and tracheal region combined [33].

**Table I:** Regional deposition of salbutamol sulphate DPI, nebuliser, and pMDI in both AIR and NGI models expressed as percentage of delivered dose. DPI and nebuliser delivery, the actuator/device was not included in the analysis, however, pMDI actuators were washed and analysed (mean ± SD, n = 3)

Device	Model	Oropharyngeal (%)	Intrathoracic (%)
DPI	AIR	94.32 ± 3.52	5.65 ± 3.49
	NGI	87.31 ± 0.49	12.69 ± 0.49
Nebuliser	AIR	19.44 ± 10.47	80.56 ± 10.47
	NGI	10.19 ± 7.83	89.81 ± 7.83
pMDI	AIR	67.44 ± 13.97	25.42 ± 10.99
	NGI	48.22 ± 4.90	44.22 ± 4.58

For nebulised salbutamol sulphate, the AIR model indicated predominant deposition in the intrathoracic region, particularly in the chamber and bottom attachments (gauze and filter). The NGI demonstrated greatest deposition on stages 3–6, also within the respirable range. Notably, oropharyngeal deposition in the AIR model was approximately two-fold higher than that measured in the NGI. For salbutamol sulphate delivered via pMDI, deposition patterns resembled those of the DPI, with the majority retained in the throat region in both platforms. However, the AIR model consistently showed greater oropharyngeal retention compared to the NGI. Within the NGI, stages 3–5 accounted for the largest fraction of drug recovery (34%), again representing respirable particles. This was higher than deposition quantified in the intrathoracic airways of the AIR model (25%) shown in Table I.

### Device Dependant Deposition Patterns

**Fig. 3:**
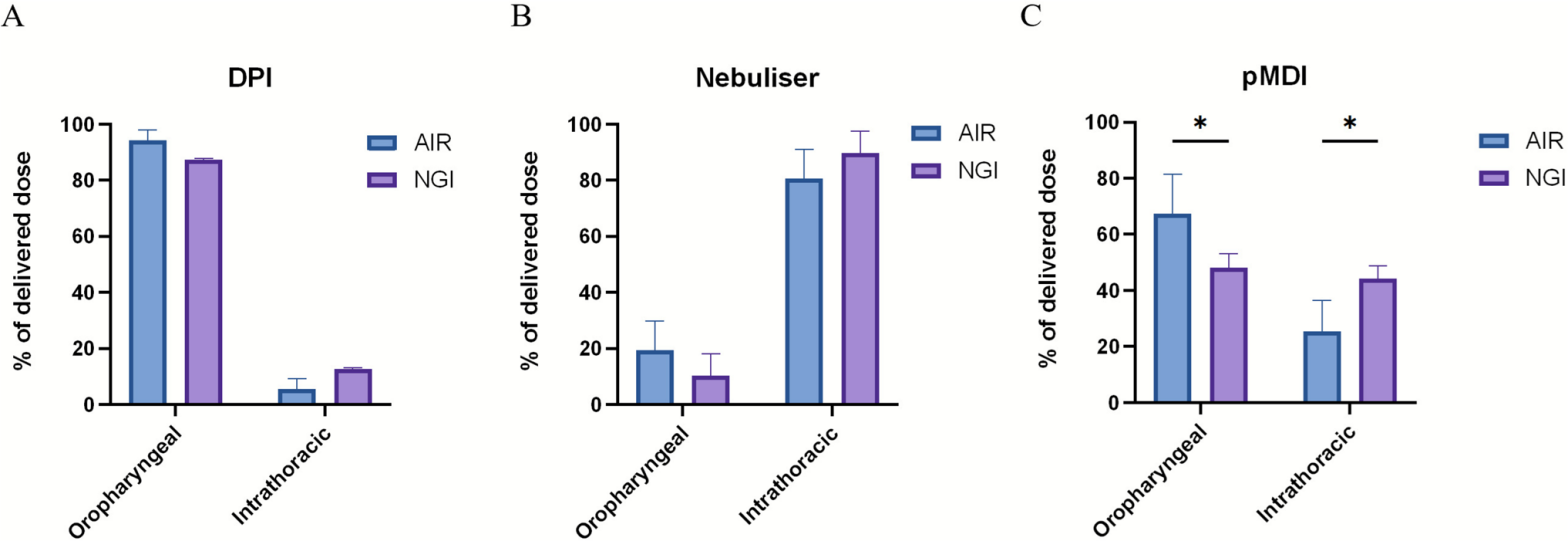
Comparative deposition of salbutamol sulphate across airway regions. Deposition in the oropharyngeal and intrathoracic regions was quantified for (**A**) dry powder inhaler (DPI), (**B**) nebuliser, and (**C**) pMDI formulations of salbutamol sulphate in both the AIR model and NGI. Data are presented as mean ± SD (n = 3). Statistical significance was assessed using two-way ANOVA with Bonferroni’s post-hoc test (*p < 0.05).

**Fig. 4:**
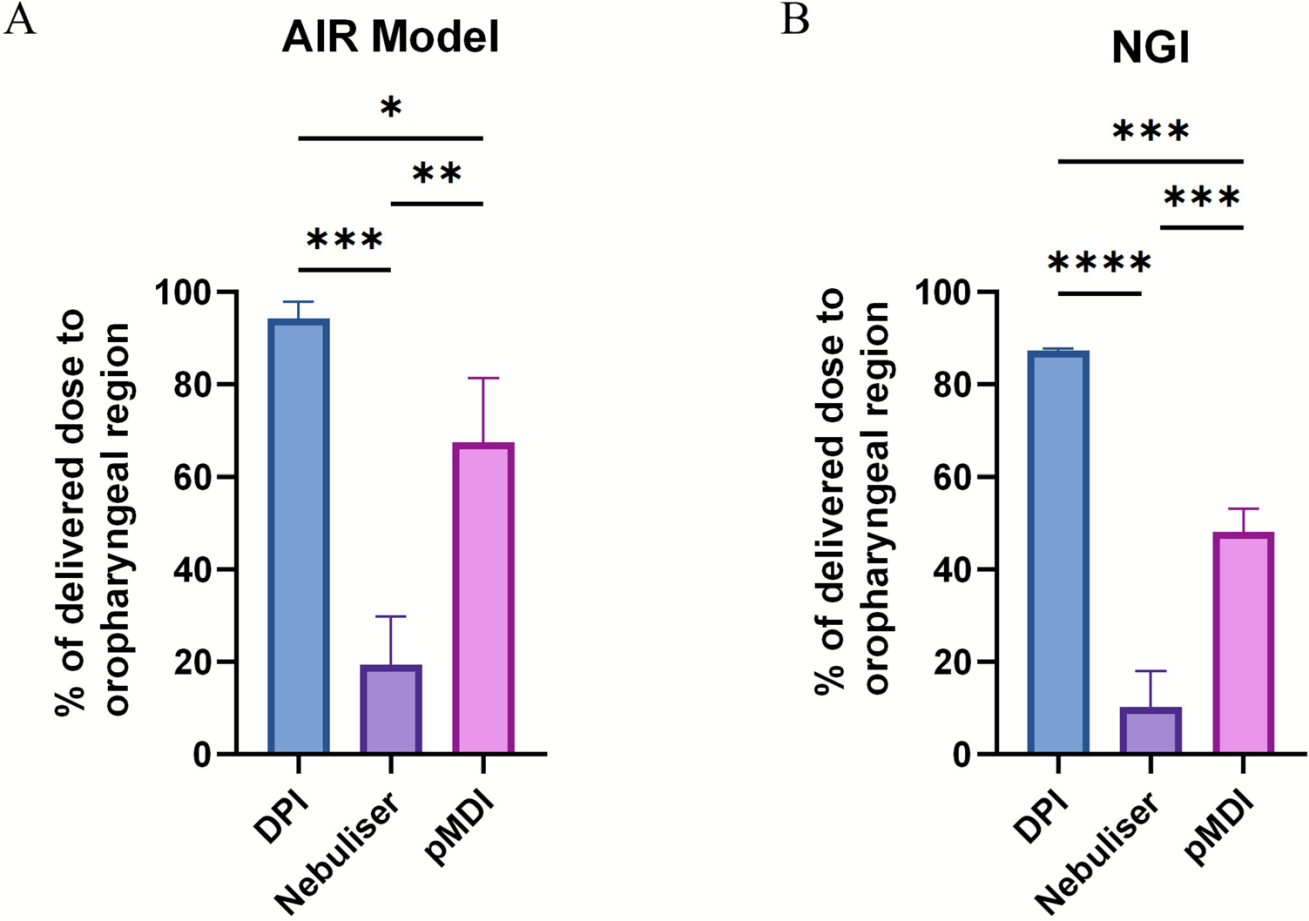
Oropharyngeal deposition of salbutamol sulphate across different formulations. Deposition in the oropharyngeal was quantified in both the (**A**) AIR model and (**B**) NGI. Data are presented as mean ± SD (n = 3). Statistical significance was assessed using one-way ANOVA with Tukey’s post-hoc test (*p < 0.05, **p < 0.01, ***p < 0.001, ****p < 0.0001).

Direct comparison of the anatomically defined regions in both models revealed distinct deposition patterns. For salbutamol sulphate delivered via DPI and nebuliser, no statistically significant (DPI: *p* = 0.076; Nebuliser: *p* = 0.083) differences were observed between the AIR model and NGI in both the oropharyngeal and intrathoracic regions, although the AIR model consistently exhibited slightly greater deposition in the oropharyngeal compartment (Fig. 3A, B). By contrast, pMDI delivery produced a significantly (*p* = 0.043) higher percentage of salbutamol sulphate in the oropharyngeal region of the AIR model compared with the NGI, with a corresponding and significant reduction in intrathoracic deposition (Fig. 3C). Comparison across delivery platforms revealed formulation-dependent differences in deposition profiles. In both the AIR model and NGI, nebulisation produced significantly lower oropharyngeal deposition of salbutamol sulphate compared with the DPI and pMDI (AIR model: p < 0.001; NGI: *p* = 0.003; Fig. 4). Furthermore, pMDI use was associated with significantly lower oropharyngeal deposition than DPI (AIR model: *p* = 0.003; NGI: *p* = 0.042; Fig. 4). These findings highlight DPI, jet nebuliser, and pMDI devices do not significantly impact the overall deposition pattern because of differences in anatomically relevant models.

## Discussion

In this study, we systematically compared the deposition profiles of salbutamol sulphate across three clinically relevant inhalation platforms (DPI, pMDI, and jet nebuliser) using the anatomically accurate AIR model and the conventional NGI. Our findings reveal that while the AIR model and NGI demonstrated consistent deposition patterns for DPI and nebuliser aerosols, pMDI aerosols were strongly influenced by anatomical complexity, resulting in markedly higher oropharyngeal deposition in the AIR model. These findings highlight the importance of incorporating anatomically representative models when evaluating device performance, particularly for propellant-driven formulations.

### Comparison of Deposition Behaviour for DPI in the AIR and NGI Model

When comparing salbutamol sulphate deposition for DPI and nebuliser platforms, no statistically significant differences (AIR model: *p* = 0.003; NGI: *p* = 0.042; Fig. 4) were observed between the AIR model and NGI in oropharyngeal or intrathoracic regions. Previous NGI studies of the Ventolin DPI (Accuhaler®) reported ~ 61% of the emitted dose retained in the oropharynx [34]. *In vivo* gamma scintigraphy in asthmatic patients shas shown 62–79% upper airway retention and 15–33% lung deposition of the total dose [35]. In our study, the Easyhaler® demonstrated higher oropharyngeal deposition (87–94%) in both AIR and NGI, likely reflecting device-specific aerodynamic properties and the influence of fixed flow rate testing (60 L/min), which may overestimate deposition relative to asthmatic patients with variable forced expiratory volume in 1 s [35]. Overall, oropharyngeal deposition of salbutamol sulphate DPI in the AIR model was 7% higher than in the NGI, with a corresponding reduction in intrathoracic deposition.

### Deposition Behaviour of Nebulised Salbutamol Sulphate in the AIR and NGI Model

For nebulised salbutamol sulphate, deposition was characterised by high recovery in intrathoracic regions and relatively low oropharyngeal retention. Nimmano *et al*. reported that only ~ 5% of salbutamol sulphate aerosolised using the PARI LC Sprint jet nebuliser at 15 L/min was retained in the oropharyngeal region, with deposition distributed primarily across NGI stages 2–5 [36]. Consistent with this, our NGI data demonstrated highest recovery in stages 3–6, with 10% deposition in the oropharynx. The AIR model quantitatively showed 9% higher oropharyngeal deposition (19%), likely due to its increased anatomical complexity compared with the USP throat, although these differences were not statistically significant (*p* = 0.832).

### Impact of Anatomical Complexity on pMDI Deposition in the AIR and NGI Model

In contrast, pMDI formulations exhibited significant differences in deposition between the AIR model and NGI. In both systems, the majority of salbutamol sulphate was retained in the oropharyngeal region, consistent with prior *in vitro* studies [13, 14]. However, the AIR model consistently exhibited significantly greater oropharyngeal deposition than the NGI (19% higher; *p* = 0.043), with a correspondingly lower intrathoracic deposition.

Wei *et al*. reported ~ 45% salbutamol sulphate (Ventolin Evohaler, with USP throat) recovery in the oropharyngeal region at 30 L/min, closely aligning with our oropharyngeal NGI deposition (~ 48%) [11]. More anatomically complex replicas, such as the Virginia Commonwealth University (VCU) human airway model, showed higher oropharyngeal deposition using the Ventolin Evohaler (74–85%, depending on size) [11]. Similarly, Cheng *et al*. observed ~ 56% oropharyngeal deposition using a human airway replica at 30 L/min with a salbutamol sulphate HFA134a commercial pMDI (Proventil HFA, Key Pharmaceuticals, Kenilworth, NJ), which compared closely with the 67% observed in the AIR model [12]. The slightly lower deposition observed by Cheng *et al*. [12] may reflect formulation differences, as Proventil HFA contains ethanol and oleic acid, excipients associated with higher total lung deposition compared with Ventolin HFA [37].

The increasing throat deposition observed across more anatomically realistic systems indicates that simplified *in vitro* models such as the NGI with USP throat, may underestimate oropharyngeal retention relative to human physiology. *In vivo* gamma scintigraphy study by Hirst *et al*., shows in healthy volunteers have reported up to 72% deposition of salbutamol sulphate from pMDIs in the oropharynx, aligning closer with AIR model outcome than that observed in the NGI shown in Table I [38]. The high-velocity plume generated by pMDIs results in greater inertial impaction in anatomically complex models with longer flow paths than in the smooth USP throat [11, 39, 40]. Overall, these comparisons highlight the critical role of airway geometry in determining deposition efficiency and with the AIR model providing estimates that more closely reflect *in vivo* observations.

### Device Dependant Trends in Regional Deposition

In this study, we observed that both the NGI and AIR models demonstrated oropharyngeal deposition in the order DPI > pMDI > nebuliser, with a corresponding inverse pattern for intrathoracic airway deposition. These differences are likely due to aerosol characteristics, particularly the lower plume velocity generated by nebulisers compared to DPIs and pMDIs, which results in reduced oropharyngeal impaction [40–42]. Previous *in vitro* and *in vivo* studies have reported mixed results regarding DPI and pMDI deposition in the oropharyngeal region, reflecting the influence of device type, inspiratory flow rate, and formulation properties [18, 35, 43]. In our study, DPI exhibited significantly higher oropharyngeal deposition than pMDI in both the AIR model and NGI. This is likely due to the high-velocity pMDI aerosol droplets undergoing rapid evaporation, making their deposition highly sensitive to airway geometry and pathlength, in contrast to DPI particles [40, 44, 45]. These results emphasise the importance of using anatomically realistic airway replicas to evaluate pMDI deposition, as traditional impactors substantially underestimate oropharyngeal deposition.

While increased oropharyngeal deposition of pMDI aerosols in anatomically realistic airway replicas has been previously reported, the present study extends this understanding in several important ways. Most existing airway models focus primarily on the mouth–throat region or simplified proximal geometries, limiting their ability to resolve how deposition in the upper airway translates to downstream intrathoracic delivery within a single experimental system. In contrast, the AIR model provides a continuous, anatomically resolved airway pathway from the oropharynx into the intrathoracic regions, enabling direct quantification of regional dose distribution without reliance on separate or sequential models.

Importantly, this study offers a systematic, head-to-head comparison of three clinically relevant inhalation platforms (DPI, pMDI, and nebuliser) tested under matched flow conditions in both an anatomically realistic model and a conventional cascade impactor. This design allows identification of when added anatomical complexity meaningfully alters deposition outcomes. Our results demonstrate that while DPI and nebuliser aerosols show comparable regional deposition between the AIR model and NGI, pMDI aerosols are uniquely sensitive to airway geometry, leading to substantially higher oropharyngeal retention and reduced intrathoracic delivery in the anatomically realistic system.

These findings suggest that the added value of anatomically realistic *in vitro* models is device-dependent rather than universal. The AIR model therefore does not aim to replace established impactor-based methods but instead provides a complementary tool that is particularly informative for propellant-driven inhalers, where simplified geometries are likely to underestimate upper airway deposition relative to *in vivo* observations.

### Relevance of Anatomically Realistic Airway Models Against Existing Models

While computational fluid dynamic (CFD) simulations can predict airway deposition, the high complexity in the underling physics and computational cost limit their suitable for routine experimental screening or formulation development [46]. *In silico* methods also lack detailed spatial and temporal detail to allow clear prediction of aerosol deposition in the respiratory tract, with only able to capture proximal airway aerosol behaviour [47]. *In vivo* scintigraphy also relies on specialised nuclear facilities, high experimental cost, and time which has a greater burden compared to traditional *in vitro* methods [26].

With the recent U.S. Food and Drug Administration (FDA) Modernization Act 2.0 and accompanying roadmaps aimed at reducing animal testing, there is an increasing emphasis on phasing out animal models and encouraging the use of *in vitro* and *in silico* systems for preclinical drug safety assessment [48]. There is a growing need for robust, human relevant *in vitro* systems that balance physiological relevance with experimental practicality. The AIR model contributes to this need by offering a reproducible, bench-top alternative capable of resolving regional deposition differences that are not captured by conventional impactor testing.

Finally, our results align with the findings of Wei *et al*., who compared deposition across eight upper airway replicas and highlighted the tendency of the USP throat to underpredict oropharyngeal retention relative to realistic airway geometries [49]. Taken together with prior computational fluid dynamics simulations and *in vivo* gamma scintigraphy studies, our data supports the use of anatomically realistic *in vitro* models, such as the AIR model, as clinically relevant tools for predicting aerosol deposition.

## Limitations

This study has several limitations that should be acknowledged. First, for pMDIs the actuator was washed and analysed, as this removable component is a recognised site of substantial drug retention. In contrast, DPIs and nebulisers do not possess comparable removable actuators; their mouthpieces are integral to the device and typically contribute minimal deposition and were therefore not analysed. While this methodological difference may introduce a minor bias, it is unlikely to alter the comparative deposition trends observed. Second, deposition testing was performed at fixed flow rates (15, 30, and 60 L/min), which may not fully capture the variability in patient inhalation profiles, particularly in individuals with compromised lung function. Third, the number of replicates was limited (n = 3), restricting statistical power to detect smaller differences. Finally, the AIR model, although anatomically detailed, represents static airflow and does not incorporate tidal breathing patterns, mucus, or humidity, which are known to influence aerosol fate *in vivo*.

Although integration of Transwell inserts has the potential to locally perturb airflow, this was mitigated in the present study by fitting pore-size matched membrane filters across all terminal outlets to equalise flow resistance and minimise branch-to-branch airflow imbalance.

Future work should address these limitations by harmonising actuator and mouthpiece analysis across devices, incorporating variable breathing profiles, and expanding replicate numbers to further strengthen the robustness of the findings.

## Conclusions

This study provides valuable insights into the differences in aerosol deposition between various salbutamol sulphate formulations in a realistic respiratory airway replica compared with a non-anatomically accurate model. We demonstrate that the physiologically relevant AIR model predicts higher oropharyngeal deposition of salbutamol sulphate delivered via pMDI compared with the NGI, indicating that pMDI aerosols are more strongly influenced by airway geometry than those from nebulisers or DPIs. Nevertheless, the overall trends in deposition patterns across formulations remained consistent between the AIR model and NGI. These findings support the complementary use of anatomically realistic and conventional models in inhaler evaluation. Future adaptations of the AIR model, including incorporation of the sinuses, artificial respiratory mucus, and nerve tissues, may further enhance its utility for advancing *in vitro* aerosol characterisation and strengthening *in vitro–in vivo* correlations.

Collectively, these findings indicate that the value of anatomically realistic *in vitro* airway models is device-dependent rather than universal, with the AIR model providing particular benefit for propellant-driven inhalers where simplified impactor geometries are likely to underestimate oropharyngeal deposition.
